# In Silico Structural Analysis of Serine Carboxypeptidase *Nf*314, a Potential Drug Target in *Naegleria fowleri* Infections

**DOI:** 10.3390/ijms232012203

**Published:** 2022-10-13

**Authors:** Pablo A. Madero-Ayala, Rosa E. Mares-Alejandre, Marco A. Ramos-Ibarra

**Affiliations:** Biotechnology and Biosciences Research Group, Faculty of Chemical Sciences and Engineering, Autonomous University of Baja California, Tijuana 22390, Mexico

**Keywords:** in silico studies, Protein Structural Analysis, serine carboxypeptidase, cathepsin A-like protein, *Naegleria fowleri*

## Abstract

*Naegleria fowleri*, also known as the “brain-eating” amoeba, is a free-living protozoan that resides in freshwater bodies. This pathogenic amoeba infects humans as a casual event when swimming in contaminated water. Upon inhalation, *N. fowleri* invades the central nervous system and causes primary amoebic meningoencephalitis (PAM), a rapidly progressive and often fatal disease. Although PAM is considered rare, reducing its case fatality rate compels the search for pathogen-specific proteins with a structure–function relationship that favors their application as targets for discovering new or improved drugs against *N. fowleri* infections. Herein, we report a computational approach to study the structural features of *Nf*314 (a serine carboxypeptidase that is a virulence-related protein in *N. fowleri* infections) and assess its potential as a drug target, using bioinformatics tools and in silico molecular docking experiments. Our findings suggest that *Nf*314 has a ligand binding site suitable for the structure-based design of specific inhibitors. This study represents a further step toward postulating a reliable therapeutic target to treat PAM with drugs specifically aimed at blocking the pathogen proliferation by inhibiting protein function.

## 1. Introduction

Carboxypeptidases are hydrolytic enzymes that cleave the C-terminal peptide bond of proteins and polypeptides, releasing free amino acid residues (usually one at a time). Furthermore, this biochemical reaction is a post-translational modification that plays a critical role in the degradation, processing, and modulation of intracellular proteins. Based on the catalytic mechanism, a typical classification sorts them into serine, metal, and cysteine carboxypeptidases [1,2,3,4].

Serine carboxypeptidases are protein components of vacuoles (plants and fungi) and lysosomes (protozoa and animal cells) [5,6,7,8,9,10,11]. Most of those studied are involved in the intracellular turnover of polypeptide substrates, but some also release amino acids from extracellular proteins or peptides [1,12,13,14,15]. Interestingly, those isolated from fungi are single polypeptides, whereas those from animal cells comprise two polypeptides linked by disulfide bonds [1,3,7,9,16,17,18].

The free-living amoeba *Naegleria fowleri* (also known as the «brain-eating» amoeba) feeds mainly on bacteria, resides in freshwater bodies, and tolerates climates up to 45 °C [19,20]. As a casual event, this pathogenic protozoan infects humans when swimming in contaminated freshwater [21]. After entering the body via the nose, *N. fowleri* invades the central nervous system and causes primary amoebic meningoencephalitis (PAM), a rapidly progressive and often fatal condition [20,21,22]. The standard drug to treat this disease is the antibiotic amphotericin B. However, the case fatality rate remains higher than 95%, even after opportune drug therapy [23,24,25].

Globally, epidemiological data underestimates *N. fowleri* infections because of the lack of accurate diagnostic tests and adequate surveillance programs [26]. In addition, efforts to develop complementary and alternative therapies are limited due to the rarity of PAM cases [20,23,24]. Nevertheless, the need for new or improved therapeutic agents capable of reducing the current case fatality rate remains compelling. In this regard, the available genomic/transcriptomic/proteomic data about *N. fowleri* provide information on specific biomolecules, e.g., novel virulence-associated gene products or well-known essential proteins, which contain structural differences from their human counterparts and that are suitable drug targets for treating PAM [27,28,29,30,31].

*Nf*314 is a serine carboxypeptidase first identified as the polypeptide encoded by a transcript differentially expressed in highly virulent cells of the *N. fowleri* LEE strain. Furthermore, increased transcript levels correlated with the ability of these amoebae to feed on mammalian cells. However, it appears to be required for pathogenesis but not for increased virulence, as brain cell-fed amoebae showed limited ability to kill mice [32]. Moreover, recent genomic and transcriptomic studies confirmed overexpression in mouse-passaged amoebic cells compared to those grown in culture [30]. Consequently, it was feasible to assume that *Nf*314 is a virulence-related protein in *N. fowleri* infections. Here, we performed a biocomputational approach to study the structural features of *Nf*314 and assess its potential as a target to discover adjuvant drugs capable of blocking amoebic infection and, thus, reducing the risk of PAM disease.

## 2. Results and Discussion

### 2.1. Nf314, a Virulence-Related Serine Carboxypeptidase

According to the original entry, *Nf*314 is a 54 kDa protein consisting of 482 amino acid residues (GenBank M88397; UniProtKB P42661). However, a search in AmoebaDB revealed a 17-residue segment missing at its N-terminus, yielding a 56 kDa polypeptide, as encoded by a single gene in two strains of *N. fowleri*: FDP41_000254 (in ATCC_30894) and NfTy_024570 (in Ty). As expected, primary structure analysis validated its secretory nature: lysosomal localization and probable export to the extracellular space. Moreover, its highly confident peptidase domain (1.4 × 10^−145^) contained the conserved catalytic triad, i.e., Ser180, Asp416, and His476 (Supplementary Figure S1), and two structural patterns linked to the active site: 176-LAGESYGG-183 and 466-LTFITVRGAGHMVPLVKP-483. Further secondary (2D) structure analysis showed that it displayed an α/β-hydrolase fold (Supplementary Figure S2) [33], typical of the S10_peptidase protein family (MEROPS classification, https://www.ebi.ac.uk/merops/; accessed on 6 June 2022) [34].

**Figure 1:**
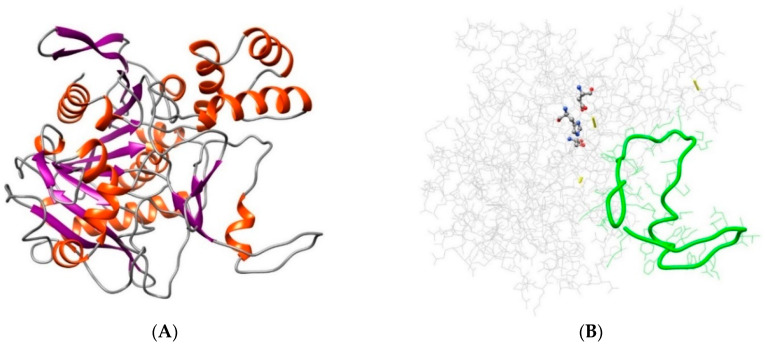
Depiction of the best 3D model for the *Nf*314 proenzyme. (**A**) Ribbon representation, colored according to the secondary structure: α-helix in red and β-sheet in purple. (**B**) Wire representation, colored in gray, with the specific location of important features (particularly highlighted): the three predicted disulfide bonds (yellow sticks), the catalytic triad (balls/sticks colored by the element), and the putative non-terminal propeptide region (tubular/wire in green).

A three-dimensional (3D) model, generated by automatic template-based prediction, revealed further insights into the tertiary structure of the *Nf*314 protein. The crystal structures of human protective protein/cathepsin A (PDB 1IVY) [35] and *Sorghum bicolor* hydroxynitrile lyase (PDB 1GXS) [36] served as suitable templates. The best 3D model (Figure 1A) displayed a good global quality score (0.695) and significant *p*-value (1.89 × 10^−8^), suggesting native-like conformation [37]. Likewise, the Ramachandran plot showed that 90.2% of the non-Gly/Pro residues were in the most favored regions, plus an additional 8.6% in allowed regions; and the estimated Z-score for overall quality, −7.62, was within the expected range for proteins of comparable size (Supplementary Figure S3). Furthermore, it appeared that three disulfide bonds stabilized the 3D structure, since six Cys residues had the spatial proximity required for chemical pairing (i.e., C_β_-C_β_ distance ≤ 4.5 Å [38]): Cys88↔Cys382, Cys248↔Cys261, and Cys284↔Cys348.

Two supplementary structural analyses detected neighboring disordered sequences located within the catalytic domain of *Nf*314. In particular, analysis of non-native 2D structures showed that the residue composition of the Lys296-Gln312 segment resembled those identified in dynamically disordered sequences (Supplementary Figure S4). Furthermore, evaluation of the 3D model quality, based on local scoring, revealed that the Leu319-Pro340 segment exhibited distances ≥4 Å between the Cα atoms in the model and their equivalents in the native structure, implying low-quality modeling, due to local intrinsic disorder (Supplementary Figure S5). Remarkably, both sequences could be portions of a putative non-terminal propeptide region (Figure 1B).

### 2.2. Nf314 Contains a Non-Terminal Propeptide Region

**Figure 2:**
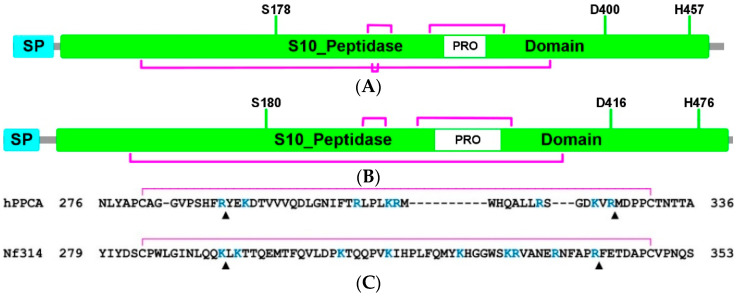
Illustration of the primary structure of hPPCA (**A**) and *Nf*314 (**B**). The signal peptide (SP, cyan) and the serine carboxypeptidase domain (S10_peptidase, green) are shown in boxes, as is the propeptide region (PRO, white) inside the latter. It also indicates the disulfide framework and the relative location of the catalytic triad. (**C**) Prediction of the *Nf*314 propeptide by sequence comparison with hPPCA. Pairwise alignment highlighting the Arg/Lys residues (R/K, blue font), usually recognized by trypsin-like proteases. The propeptide processing sites, identified for hPPCA and predicted for *Nf*314, are indicated with black triangles (up-pointing). The disulfide bond opposite and adjacent to the PRO region is also delineated (magenta line).

A comparative structural analysis, using human protective protein/cathepsin A (hPPCA) as a template, supported the hypothesis that a putative disordered region was the propeptide of *Nf*314 (Figure 2). This theoretical approach also provided further insight into the most probable proteolytic cleavage sites for its processing, a post-translational modification required for enzyme maturation.

The hPPCA protein consisted of 480 amino acid residues comprising a signal peptide and a serine carboxypeptidase domain stabilized by four disulfide bonds (Figure 2A). In vivo, it was produced as a preproenzyme and transported via the secretory pathway to the lysosome. However, before reaching this compartment, its signal peptide was cleaved, and the resulting proenzyme remained inactive, due to a propeptide that blocks the catalytic site. Once inside the lysosome, this structural lid was processed by trypsin-like proteases, relieving inhibition and producing the mature/active hPPCA enzyme, which consisted of two polypeptide chains (32 and 20 kDa) linked by disulfide bonds [7,35,39]. Remarkably, a comparison of the primary structure showed that the *Nf*314 protein (Figure 2B) and hPPCA shared significant similarities, e.g., the relative location of the catalytic triad and the propeptide region. Moreover, a local pairwise sequence alignment revealed putative cleavage sites for trypsin-like proteases, including those potentially involved in *Nf*314 propeptide processing: Lys294↓Leu295 and Arg341↓Phe342 (Figure 2C). Based on these findings, it seemed rational to propose that the polypeptide sequence extending from Leu295 to Arg341 was the propeptide of *Nf*314, which might be the structural modulator of protein function (blocking the catalytic site and, thus, preventing uncontrolled enzyme activity during the inactive state [40,41]), and had to be further processed by trypsin-like proteases to obtain the mature protein.

### 2.3. Mature Nf314 Exhibits a Ligand Binding Site

**Figure 3:**
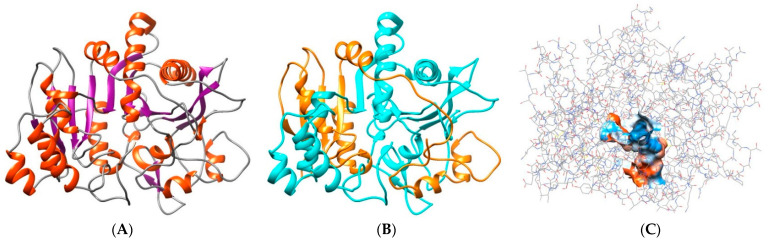
Depiction of the best 3D model for the mature *Nf*314 enzyme. Ribbon representation, colored according to (**A**) secondary structure, α-helix in red and β-sheet in purple), or (**B**) polypeptide chains produced after putative propeptide processing, large in cyan and small in orange. (**C**) Wire representation, colored by the element, highlighting the specific location of the predicted ligand-binding pocket (surface colored by hydrophobicity).

A computational approach comprising manual editing (i.e., propeptide removal) of the previously generated 3D model, followed by structural stabilization using molecular dynamics (MD) simulations, produced a consistent model for the mature *Nf*314 enzyme, as confirmed by a structural quality analysis: 93.6% of the residues scored acceptable values (≥0.2) in the 3D/1D correlation profile. Moreover, its ligand binding site showed significant overlap with counterparts of similar proteins whose 3D structure has been solved, including the most probable ligand-contacting residues and the protein–ligand interaction pocket (Figure 3).

### 2.4. Nf314 Has Potential as a Drug Target

Three β-amino acid derivatives, identified as hPPCA inhibitors [42], named **2a**, **8a**, and **15a** (Supplementary Figure S6), functioned as the test ligands to assess the potential of *Nf*314 as a drug target. Remarkably, in silico molecular docking experiments and MD simulations revealed that all ligands formed stable complexes with the receptor, showing plausible binding energies (Table 1). Moreover, the latter values were equivalent to those calculated for the hPPCA–ligand complexes: −8.6, −8.8, and −8.4 (in kcal/mol) for **2a**, **8a**, and **15a**, respectively. Therefore, it is worth noticing that this remark indirectly validated our approach and the resulting data, as both estimations applied inputs from the same biocomputational protocol.

**Figure 4:**
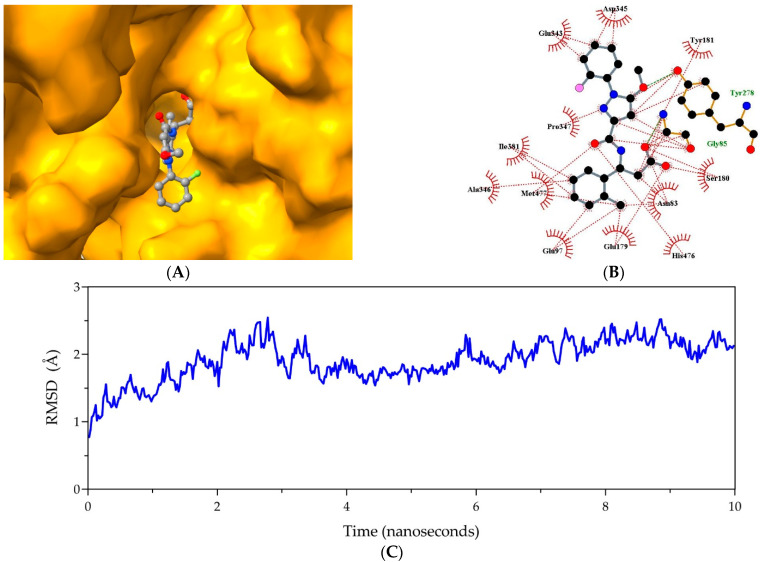
Structural analyses of the *Nf*314-2a complex. (**A**) Illustration of the best binding pose of *Nf*314 (orange surface) and compound **2a** (balls/sticks colored by the element). (**B**) 2D representation of the *Nf*314-2a non-covalent interaction network. Colors: hydrogen bonds, green dashes; hydrophobic interactions, red dashes/arcs; carbon, black; oxygen, red; nitrogen, blue; fluorine, pink; ligand bonds, gray; protein bonds, orange. (**C**) Depiction of the root mean square deviations (RMSDs) trajectory, colored in blue, during MD simulations performed to assess the *Nf*314-2a complex stability.

In particular, compound **2a** (also known as SAR164653 or SAR1) is regarded as the first-line inhibitor of hPPCA because it successfully passed early phase clinical trials, showing a favorable safety profile in healthy human subjects [43], and has potential as a heart failure attenuating drug in post-myocardial infarction treatment [44]. Based on this information, it seems reasonable to propose that compound **2a** represents a reliable lead for *Nf*314-specific drug design. Furthermore, detailed structural analyses supported the latter suggestion. For instance, the most stable complex showed that compound **2a** posed inside the ligand-binding pocket of *Nf*314 (Figure 4A), establishing a significant number of non-covalent interactions (Figure 4B), and remained bound to *Nf*314 under solvated conditions and unrestricted protein dynamics (Figure 4C). Moreover, it exhibited moderate binding affinity (i.e., a free energy of −1.6 kcal/mol).

Overall, the other two ligands (compounds **8a** and **15a**) showed valuable structural features as well (Supplementary Figure S7), so they could be useful as support molecules in a broad and exhaustive approach for the *Nf*314-specific drug design, ensuring target selectivity and, thus, reducing the risk of potential side effects associated with inhibition of hPPCA and other human proteases.

## 3. Materials and Methods

### 3.1. Sequence Retrieval and Database Searching

The polypeptide sequence of *Nf*314 (deduced from a virulence-related transcript in *N. fowleri* LEE strain [32]) was retrieved from UniProtKB (https://www.uniprot.org/; accessed on 14 June 2022) [45], using the accession number P42661. This sequence was later used as a query to identify the complete sequence by BLAST search in AmoebaDB (https://amoebadb.org/; accessed on 14 June 2022) [46,47], which contains the genomic databases of three *N. fowleri* strains: ATCC_30863, ATCC_30894, and Ty.

### 3.2. Primary and Secondary Structure Analyses

The primary physicochemical parameters were determined using the Expasy ProtParam tool (https://web.expasy.org/protparam/; accessed on 15 June 2022) [48]. The conserved, potentially functional serine carboxypeptidase domain was delimited using the NCBI CD-Search tool (https://www.ncbi.nlm.nih.gov/Structure/cdd/; accessed on 15 June 2022) [49,50], whereas the protein architecture was defined using the database services of InterPro (https://www.ebi.ac.uk/interpro/; accessed on 20 June 2022) [51], Pfam (https://pfam.xfam.org/; accessed on 20 June 2022) [52], and SMART (http://smart.embl-heidelberg.de/; accessed on 20 June 2022) [53]. Protein sorting signals and subcellular localization were verified using SignalP, TargetP, and DeepLoc (https://services.healthtech.dtu.dk/; accessed on 20 June 2022) [54,55,56]. The secondary structure, and the putative disordered regions, were predicted using the PSIPRED workbench (http://bioinf.cs.ucl.ac.uk/psipred/; accessed on 21 June 2022) [57]. Orthologous proteins were detected using the NCBI BLAST search engine (https://blast.ncbi.nlm.nih.gov/; accessed on 21 June 2022) [58]. Unless otherwise specified, all multiple sequence alignments were generated using the EBI Clustal Omega tool (https://www.ebi.ac.uk/Tools/msa/clustalo/; accessed on 21 June 2022) [59].

### 3.3. Template-Based Modeling and 3D Structure Validation

A 3D model for the *Nf*314 proenzyme was produced by automatic template-based prediction using IntFOLD (https://www.reading.ac.uk/bioinf/IntFOLD/; accessed on 23 June 2022) [37]. The output of this server included estimation of model accuracy and identification of ligand binding residues applying the ModFOLD [60] and FunFOLD [61] methods. The PROCHECK (https://saves.mbi.ucla.edu/; accessed on 25 June 2022) [62] and ProSA (https://prosa.services.came.sbg.ac.at/; accessed on 25 June 2022) [63] evaluation tools further validated the tertiary structure.

The best representative 3D structure for the mature *Nf*314 enzyme was generated by computer-aided removal of the propeptide and structural stabilization through MD simulations, using the proenzyme model as a starting conformation. The computational resources of VMD [64] and QwikMD [65] were combined to get a stable 3D structure under solvated conditions. Two consecutive 30-ns simulations were performed at 25 °C on NPT systems with explicit solvent and standard settings for minimization, annealing, and equilibrium. Briefly, the first one refined and stabilized the newly generated free-end residues, and the 3D conformation from a cluster analysis served as a source for the second one, conducted without any constraints. Lastly, a further cluster analysis worked to select the best 3D model, validated by the online structural quality assessment tool VERIFY3D [66], available at SAVES (https://saves.mbi.ucla.edu/; accessed on 26 June 2022).

The protein tertiary structure was analyzed using the UCSF Chimera and ChimeraX as interactive systems for molecular graphics [67,68].

### 3.4. Analysis of the Protein-Ligand Binding Site

The ligand-binding site of the mature *Nf*314 enzyme was predicted using the online computational services of FunFOLD (https://www.reading.ac.uk/bioinf/FunFOLD/; accessed on 3 July 2022) [61] and COACH (https://zhanggroup.org/COACH/; accessed on 3 July 2022) [69,70]. The output of these web tools, i.e., the most probable ligand-contacting residues and the protein–ligand interaction pocket, were combined and filtered, and the resulting ligand-binding site was validated using CASTp (http://sts.bioe.uic.edu/castp/; accessed on 3 July 2022) [69]. The topography of the ligand binding surface was visualized using UCSF Chimera.

### 3.5. In Silico Molecular Docking Experiments

The 3D structure of mature *Nf*314 was used as a receptor for in silico molecular docking experiments of three β-amino acid derivatives (ligands), using the AutoDock Vina [71] and MGLTools [72] software with standard conditions for automated receptor–ligand binding prediction. Briefly, a grid box was defined in the receptor (based on the 3D conformation of its binding site) and used as the target for docking, assuming a rigid receptor and a flexible ligand. Moreover, independent standard MD simulations were performed to assess the stability of the resulting receptor–ligand complex. The three best clusters from each simulation were analyzed using UCSF Chimera. Theoretical binding energy was estimated using the docking software default settings. In addition, the CaFE tool was employed to calculate the binding affinity, with molecular mechanics Poisson–Boltzmann surface (MM/PBSA) as the end-point method [73].

## 4. Conclusions

The high case fatality rate of human primary amoebic meningoencephalitis (PAM), a rapidly progressive disease caused by accidental infection with the free-living amoeba *N. fowleri*, and the current limited availability of effective drugs against the pathogen, compel the search for specific therapeutic targets (e.g., virulence-associated proteins) leading to the discovery of novel, or improved, drugs with potential application in the medical treatment of PAM disease. Considering the latter premises as sound arguments, we performed a biocomputational approach to study the serine carboxypeptidase *Nf*314 and evaluate its prospects as a drug target. Our findings reveal that its ligand-binding site displays a conformation amenable to interacting with specific molecules, e.g., it forms stable receptor–ligand complexes with three β-amino acid derivatives that have shown pharmacological inhibition of the human homolog. Moreover, these derivatives could be useful as lead molecules for the rational design of structure-based *Nf*314-specific inhibitors. Although experimental studies are required to validate the protein structure and characterize its enzyme activity, including inhibition by compound **2a**, this in silico structural approach is the first step toward recognizing this virulence-related protein as a target for the discovery of new drugs with the potential to block *N. fowleri* infection and, thus, its progression to PAM disease.

## Figures and Tables

**Table 1:** Calculated binding energies (kcal/mol) from the three best clusters of each *Nf*314-ligand complex.

Cluster	*Nf*314-2a	*Nf*314-8a	*Nf*314-15a
1	−8.0	−8.1	−8.0
2	−8.0	−8.3	−8.3
3	−7.4	−7.9	−7.1

## Data Availability

All relevant and supporting data, except nucleotide and polypeptide sequences, are included in this paper and the supplementary materials document.

## Supplementary Materials

The following supporting information is available (downloadable) at: https://www.mdpi.com/article/10.3390/ijms232012203/s1.
