# Supplementary material for: In Silico Structural Analysis of Serine Carboxypeptidase Nf314, a Potential Drug Target in Naegleria fowleri Infections
Source: Int J Mol Sci. 2022 Oct 13;23(20):12203. doi: 10.3390/ijms232012203 (PMC9603766; doi:10.3390/ijms232012203)
Supplement: Supplementary file 1 [file ijms-23-12203-s001.zip › ijms-1924927-supplementary.pdf]

International Journal of Molecular Sciences

Supplementary Material

In Silico Structural Analysis of Serine Carboxypeptidase *Nf*314, a Potential Drug Target in *Naegleria fowleri* Infections

Pablo A. Madero-Ayala, Rosa E. Mares-Alejandre and Marco A. Ramos-Ibarra *

Biotechnology and Biosciences Research Group, Faculty of Chemical Sciences and Engineering, Autonomous University of Baja California, Tijuana, Mexico.

* Correspondence: mramos@uabc.edu.mx

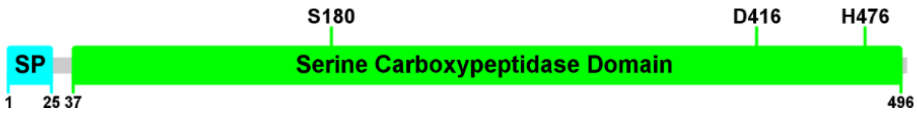

Figure S1. Illustration of the primary structure of *Nf*314 protein. The signal peptide (SP, cyan) and peptidase domain (green) are shown in rectangles with their boundaries at the bottom. The specific location of the catalytic triad is also indicated (top of peptidase domain). A remark on UniProtKB P42661: the sequence appears incomplete (i.e., 17 residues are missing at the N-terminus); hence the peptidase domain locates the catalytic triad at positions 163, 399, and 459.

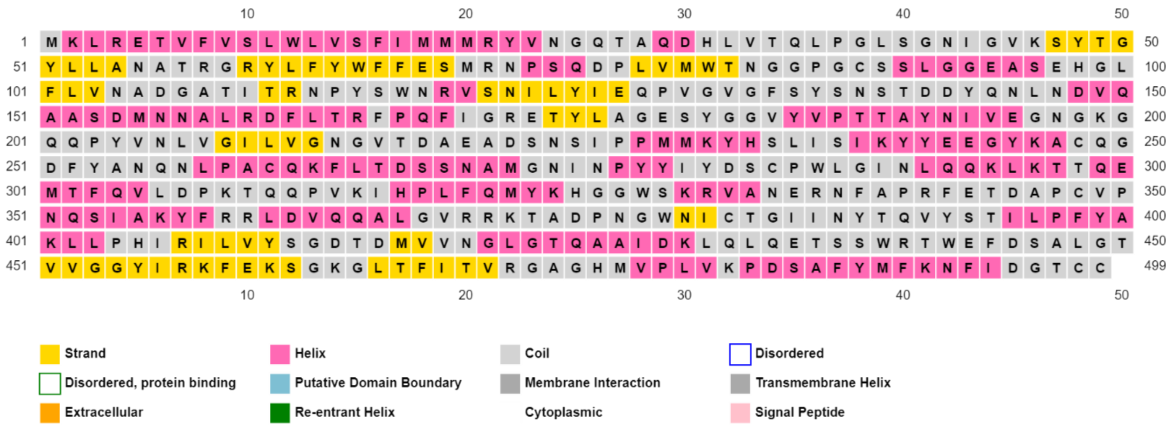

Figure S2. Secondary (2D) structure prediction of the *Nf*314 protein using the PSIPRED workbench. Graphical output of the online 2D-specific service (http://bioinf.cs.ucl.ac.uk/psipred/). Colors used for sequence annotation are shown at the bottom (default settings).

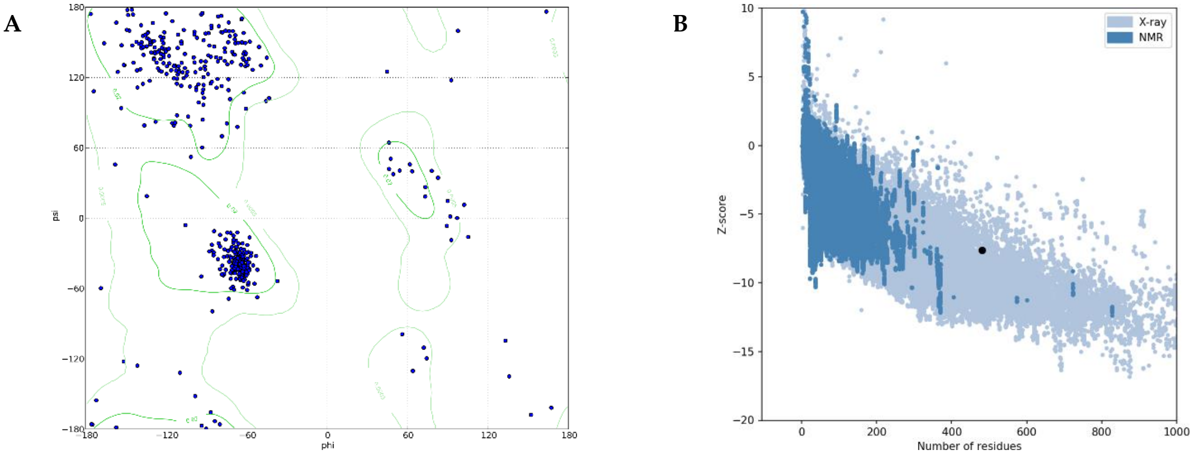

Figure S3. Quality assessment of the predicted tertiary structure of *Nf*314 protein. Ramachandran (**A**) and Z-score (**B**) plots of the best three-dimensional (3D) conformation generated by template-based modeling. The black dot in (B) denotes the estimated Z-score for *Nf*314.

Figure S4. Graphical representation of confidence estimates for the structural disorder, predicted by DISOPRED3 (http://bioinf.cs.ucl.ac.uk/psipred/), against the relative position of each residue in the *Nf*314 protein sequence. The dashed line marks the threshold (≥ 0.5) and defines the disordered region (red stripe).

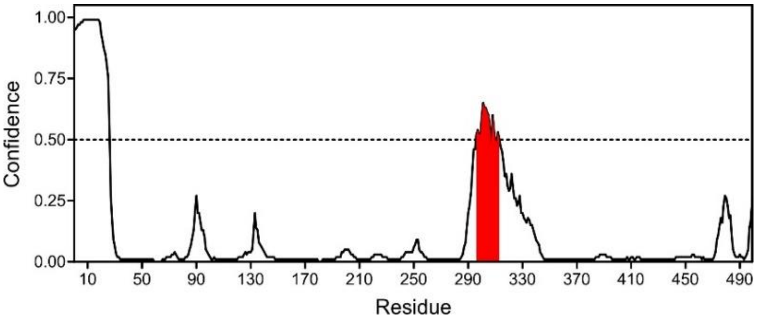

Figure S5. Graphical representation of the local structural quality, predicted by ModFOLD (https://www.reading.ac.uk/bioinf/ModFOLD/), against the relative position of each residue in the *Nf*314 protein sequence. The dashed line marks a typical threshold (≥ 4 Å) and defines the disordered region (red stripe).

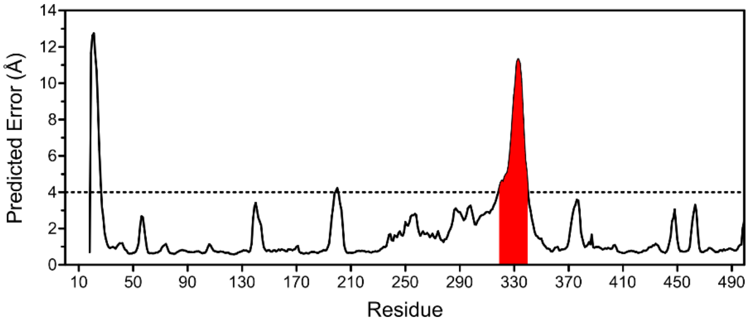

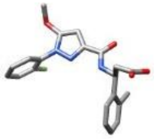

2a
IC_{50} = 26 nM

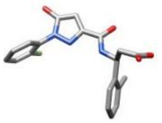

8a
IC_{50} = 38 nM

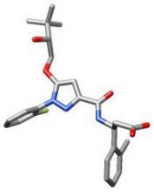

15a
IC_{50} = 5 nM

Figure S6. 3D structure of the β-amino acid derivatives used to assess the potential of *Nf*314 as a drug target. Compounds **2a**, **8a**, and **15a**, as reported in PMID 22861813 (doi: 10.1021/jm300663n). The IC_{50} values (at the bottom) represent the compound's efficacy on hPPCA.

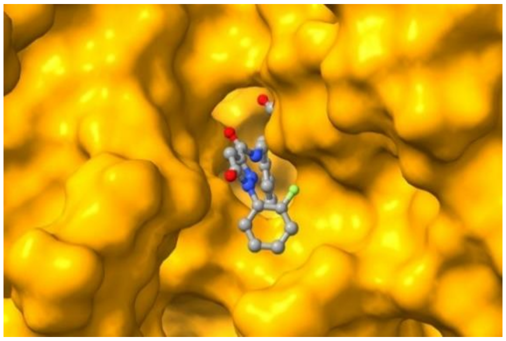

A

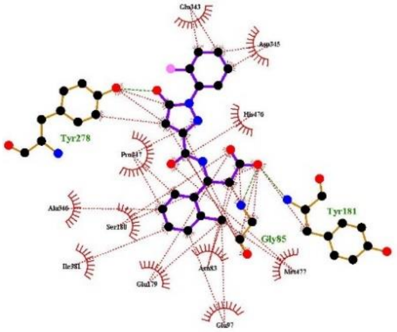

C

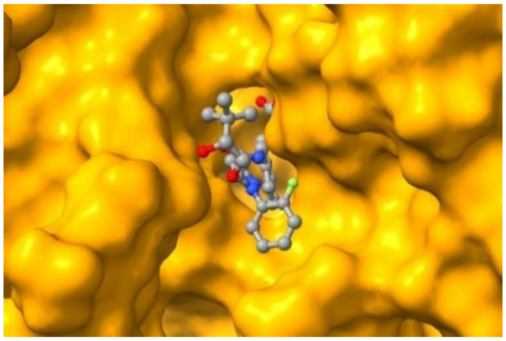

B

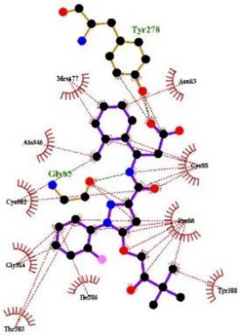

D

Figure S7. The *Nf*314-8a and *Nf*314-15a binding complexes. Best-pose depiction of *Nf*314 (orange surface) with compounds 8a (**A**) and 15a (**B**) (element-colored balls and sticks). 2D representation of the non-covalent interaction networks of *Nf*314-8a (**C**) and *Nf*314-15a (**D**). Colors: hydrogen bonds, green dashes; hydrophobic interactions, red dashes/arcs; carbon, black; oxygen, red; nitrogen, blue; fluorine, pink; ligand bonds, gray; protein bonds, orange.
